# Endobronchial Lipomatous Hamartoma: An Incidental Finding in a Patient with Atrial Fibrillation—A Case Report

**DOI:** 10.1155/2012/897581

**Published:** 2012-02-02

**Authors:** Friederike Schneider, Hauke Winter, Florian Schwarz, Manuel Niederhagen, Vivian Arias-Herrera, Eimo Martens, Stefan Kääb, Hans Theiss

**Affiliations:** ^1^Department of Internal Medicine III, University Hospital Munich, Campus Grosshadern, Marchioninistr. 15, 81377 Munich, Germany; ^2^Department of Surgery, University Hospital Munich, Campus Grosshadern, Marchioninistr. 15, 81377 Munich, Germany; ^3^Department of Radiology, University Hospital Munich, Campus Grosshadern, Marchioninistr. 15, 81377 Munich, Germany; ^4^Department of Pathology, University Hospital Munich, Campus Grosshadern, Marchioninistr. 15, 81377 Munich, Germany; ^5^Department of Internal Medicine I, University Hospital Munich, Campus Grosshadern, Marchioninistr. 15, 81377 Munich, Germany

## Abstract

*Introduction*. Lung hamartomas are the most common benign tumors of the lung. Typically, they are located in the peripheral lung, while an endobronchial localisation is rare. *Case Presentation*. We present a case with the rare diagnosis of an endobronchial hamartoma as incidental finding in a 69-year-old male, caucasian patient with atrial fibrillation. At first admission, the patient's exertional dyspnea was caused by atrial fibrillation. Relapse of exertional dyspnea in the absence of arrhythmia was due to postobstructive pneumonia caused by an endobronchial hamartoma. *Conclusion*. Endobronchial tumors such as endobronchial lipoma or hamartoma should be considered as potential causes of exertional dyspnea and thus as differential diagnosis of atrial fibrillation. Although endobronchial hamartomas are benign, resection is recommended to prevent postobstructive lung damage.

## 1. Introduction

Hamartoma is the most common benign pulmonary tumor. Endobronchial lipomatous hamartoma is a rare benign tumor of the endobronchial tree. It is often diagnosed late when postobstructive complications, such as pneumonia and bronchiectasis, and irreversible pulmonary damage have already occurred.

## 2. Case Presentation

A 69-year-old patient was admitted to the hospital with exertional dyspnea and shortness of breath. He denied cough or hemoptysis and negated questions for nightsweats, fever, or weight loss. The patient had never smoked.

Routine laboratory workup was normal. Physical examination revealed slightly diminished breath sounds in the right basal lung field. The chest scan showed a prominent right hilum, but no pleural effusion or pulmonary infiltrates were noted. Pericardial effusion was ruled out by cardiac ultrasound.

**Figure 1:**
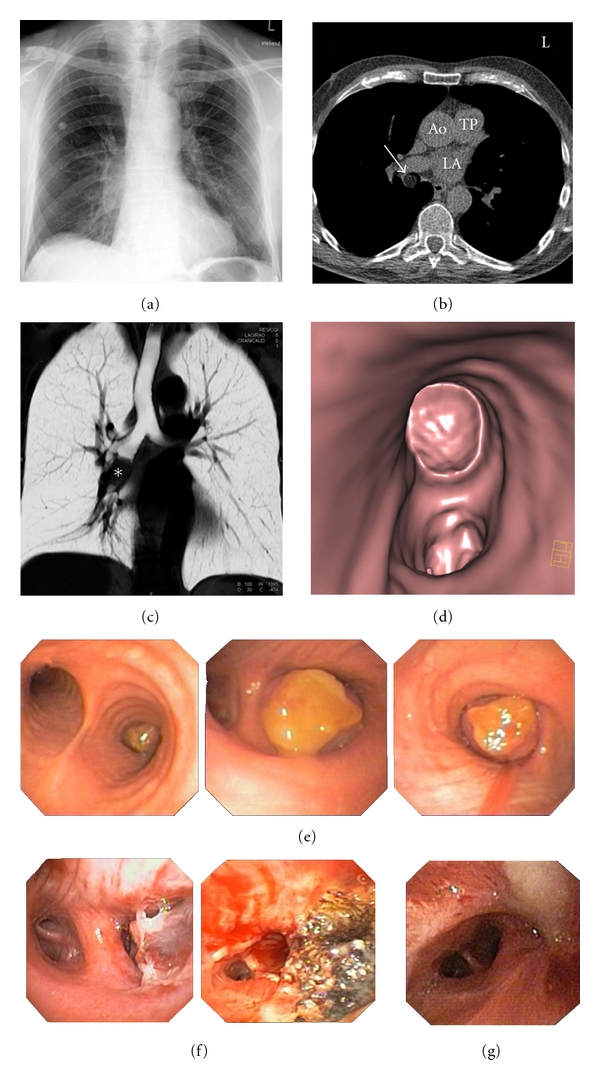
(a) On p.a. chest X-ray a prominent right hilum and a subtle increase in parenchymal density in the right infrahilar region was noted, a mass could not be clearly identified, however. (b) A noncontrast-enhanced CT scan of the chest revealed a mass within the bronchus intermedius (arrow) consisting mostly of fat with minimal inclusions of soft tissue density (Ao: Aorta ascendens; PT: pulmonary trunk; LA: left atrium). (c) Coronal slide in inverted thin-volume-rendering-technique of a contrast-enhanced CT scan performed for further workup demonstrated position and extent of the mass within the bronchus intermedius. (d) Virtual-bronchoscopy reconstruction from the nonenhanced CT scan displayed the mass within the proximal bronchus intermedius. The adjacent upper lobe bronchus was not affected. (e) Endobronchial round tumorous obstruction of the intermediate bronchus with a smooth surface with subtotal obstruction of the intermediate bronchus. (f) Recurent subtotal exclusion of the intermediate bronchus in the course of the disease and repetition of Argon laser therapy. (g) Incipient focal metaplasia of respiratory mucosa into squamous epithelium.

Electrocardiogram showed persistent atrial fibrillation (AF). Previously, the patient had undergone various therapies for AF including recurrent electric cardioversions and catheter ablation of the pulmonary veins. Thus, dyspnea was initially thought to be related to AF, and reisolation of the pulmonary veins was performed successfully. To screen for coronary artery disease prior to the administration of a class I antiarrhythmic drug as relapse prophylaxis, a noncontrast-enhanced, ECG-gated Multidetector CT (MDCT) scan of the heart was performed. Presence of relevant coronary calcium was excluded, but MDCT revealed a round mass of 1.9 cm diameter with fat equivalent CT density values in the right intermediate bronchus as an incidental finding (Figures 1(a)–1(d)). There was no contrast enhancement or popcorn calcification of the tumor. Lymph nodes were not enlarged. Since dyspnea improved after conversion into the sinus rhythm, the patient was discharged and referred for further workup of this finding on an outpatient basis. 

On a follow-up visit one month later, he again complained of dyspnoea on exertion. This time, AF was ruled out. A contrast-enhanced MDCT scan of the chest was performed and revealed a beginning middle lobe pneumonia.

Flexible bronchoscopy detected a round tumorous lesion with a smooth surface without hypervascularization subtotally occluding the intermediate bronchus. The intermediate bronchus could be passed by the bioptic gripper and brush only, but not with the bronchoscope itself (Figures 1(e)–1(g)). Microbiological analysis of the bronchoalveolar lavage revealed E.coli, thus therapy with amoxicillin/clavulan acid was initiated.

Histopathological examination of the biopsies showed benign bronchial mucosa of the intermediate bronchus with edema, fibrosis, and chronic inflammation. There was no evidence for malignancy, carcinoid tumor, granulomatous inflammation, or acid fast rods. In deeper layers, mature fatty tissue could be detected marginally, suggesting a lipoma or lipomatous hamartoma. The endobronchial tumor was removed to 95% by loop excision and laser therapy (9000J) in a second rigid bronchoscopy. Argon laser therapy was repeated once due to extensive mucous retention and subtotal occlusion of the intermediate bronchus. The patient was discharged from the hospital in good performance status one week later. Initially, follow-up bronchoscopies were performed monthly. Due to progredient granulation and occlusion of the middle lobe, endoluminal laser therapy was repeated twice (5500J), two and three months later.

Recurrent obstructions of the middle lobe combined with incipient focal metaplasia of respiratory mucosa into squamous epithelium indicated surgical sleeve resection of the middle lobe, which was successfully performed six months after first diagnosis. Serum tumor markers CEA, CYFRA 21-1, NSE, and SCC were normal, Pro GRP (40 pg/mL) was slightly elevated.

**Figure 2:**
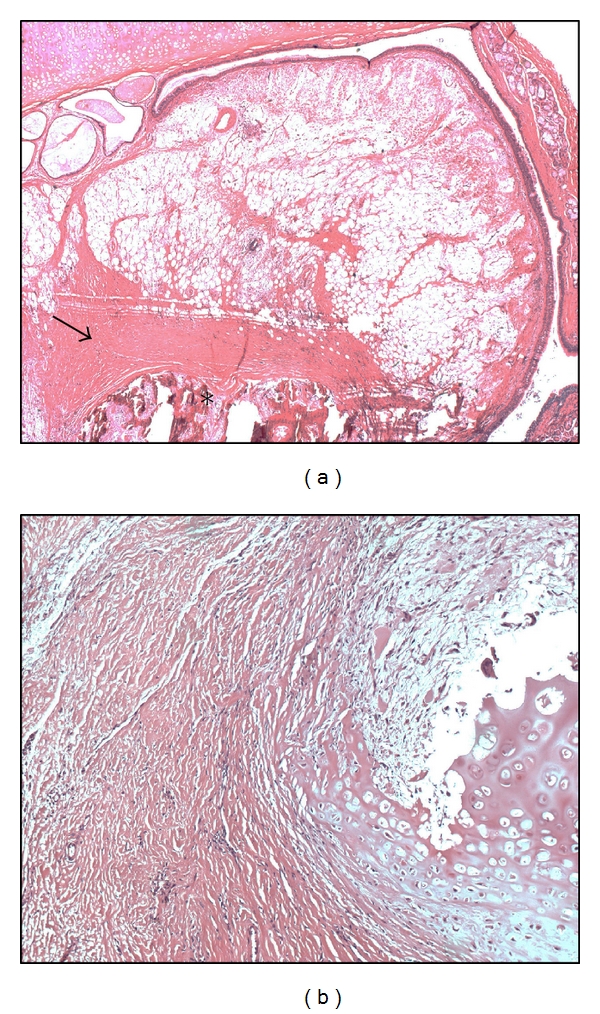
(a) Part of the hamartoma extending polyploidly into the endobronchial lumen with transition in the more spindle-celllike (arrow) and osseous component (star) (HE, 25-fold magnification). (b) Further components of the hamartoma with chondroid parts (B, HE, 100-fold magnification) within a cell-reduced, partly fascilular stroma with a hemangio pericytoma-like vessel pattern.

Histological examination of the resected tissue showed a mature benign tissue consisting of different components in accordance with the diagnosis of an endobronchial hamartoma (Figure 2). After surgical resection, the patient's dyspnea and constitution improved rapidly.

## 3. Discussion

Lung hamartomas are the most common benign pulmonary tumors (incidence between 0.025% and 0.32% [1]), mostly localized in the peripheral lung. In one large retrospective observational case study, the relative frequency of endobronchial hamartomas was found to be rare since they accounted for only 1.4% of pulmonary hamartomas [2]. In contrast, older studies have reported a considerably higher frequency of endobronchial hamartomas, ranging between 8–20% of all pulmonary hamartomas [3–5].

Endobronchial hamartomas originate from the bronchus and may contain components of mature cartilage, muscle, fat, fibrous tissue, and epithelial components. Typically, endobronchial hamartoma contains more fat than parenchymal hamartoma [6].

Middle/old-aged (5th–7th decade) men are predominantly affected with a male : female ratio of 3–5 : 1 [7]. The majority (>80%) of patients with hamartomas are smokers [2].

Although endobronchial hamartoma is a benign tumor, early diagnosis and therapy are necessary to prevent postobstructive lung damage and preserve distal lung function. Clinical symptoms often occur late [2] in dependency on the degree of airway obstruction and cannot be distinguished from other causes of endoluminal obstruction. Common symptoms include cough, wheezing, and intermittent shortness of breath leading to misdiagnoses of asthma or chronic obstructive pulmonary disease [8, 9]. Hemoptysis can occur in patients with lung hamartomas, while endobronchial lipomas are not vascularized and thus are not typically associated with hemoptysis (unless as a symptom of a postobstructive infection).

Sensitivity of chest X-ray in the diagnosis of endobronchial tumors is low (66%). Findings are often nonspecific and related to postobstructive changes such as pleural effusions and atelectasis in symptomatic patients and enlarged hila, parenchymatous consolidation, and bronchiectasis in asymptomatic patients. MDCT and MRI can narrow differential diagnoses to endobronchial lipomatous hamartoma or endobronchial lipoma if the tumor contains fatty tissue. CT then typically shows a fatty lesion with a density between 70 HU–140 HU without contrast enhancement. Whereas endobronchial lipoma shows a homogenous fat density, tissue density is more heterogenous in hamartoma and might show additional calcification in up to one third of hamartomas [6, 10]. Similar results can be obtained by MRI [11]. Bronchoscopy is indispensable for differentiation from malignancy and inflammation.

Early resection of benign endobronchial tumors may avert significant morbidity and prevent distal lung damage. The method of resection (surgical versus bronchoscopic) depends on operability of the patient, tumor size, and the degree of lung damage. 

Bronchoscopic resection and electrosurgery by Argon plasma (APC) and YAG laser can achieve complete resolution of symptoms with low interventional risk compared to surgery [12]. Completely resected endobronchial hamartoma has a low rate of recurrence [7]. Since endobronchial resection is often not complete due to tumor growth into the bronchus wall, relapses can occur after endobronchial resection. If the tumor is diagnosed late and extended irreversible lung damage has already occurred or if tumor dignity is uncertain, a surgical approach (pneumonectomy, lobectomy) is the therapy of choice. Prognosis of endobronchial hamartoma is good. Most hamartomas grow slowly, and risk of malignancy is low [7]. Nevertheless, cytogenetic studies have identified chromosomal recombinations 6p21 and 14q24 supporting that hamartomas can be clonal diseases. In single cases, endobronchial hamartoma can be transformed into malignant sarcoma [13].

## 4. Conclusion

In our male, middle-aged patient the diagnosis of endobronchial hamartoma was made incidentally by CT screening for coronary calcium. Initially, the symptom of exertional dyspnea was related to AF and improved after conversion to sinus rhythm. On second admission due to reoccurrence of exertional dyspnea, CT scan showed a beginning pneumonia caused by an endobronchial tumor leading to postobstructive infection as differential diagnosis for the dyspnea.

Initial histology from bronchoscopy revealed an endobronchial lipoma, whereas final histology performed in material from the complete surgical resection showed a hamartoma. Both tumors are mesenchymal tumors and since hamartomas may contain fatty tissue, material for first histology might only have included the fatty layer of the original hamartoma. Finally, surgical resection significantly improved the physical constitution of the patient.

Taken together, endobronchial tumors such as endobronchial lipoma or hamartoma should be considered as rare differential diagnosis of exertional dyspnea that can lead to permanent lung damage if not diagnosed and treated at an early stage.

Our case report could teach an important lesson in primary patient care. Recurrent patient complaints are often attributed to a previously established diagnosis such as the link between atrial fibrillation and dyspnoea in our case report. Nevertheless, if previous etiology can be ruled out or can just explain in part the severity of the recurrent symptom, it is important to consider other differential diagnoses.
